# Clinical features and outcomes in kidney transplant recipients with COVID-19 pneumonia: a single center retrospective cohort study

**DOI:** 10.3389/fcimb.2024.1392491

**Published:** 2024-08-15

**Authors:** Liang Xu, Xiuxiu Chen, Xuying Yang, Song Chen, Meng Yang, Zehua Yuan, Rentian Chen, Jianli Wang, Hongtao Jiang, Jian Xu, Yi Wang

**Affiliations:** ^1^ Department of Organ Transplantation, the Second Affiliated Hospital of Hainan Medical University, Haikou, China; ^2^ Department of Breast and Thyroid Surgery, the Second Affiliated Hospital of Hainan Medical University, Haikou, China; ^3^ Department of Scientific Affaires, Hugobiotech Co., Ltd., Beijing, China; ^4^ Department of Nephrology, Hainan Affiliated Hospital of Hainan Medical University, Haikou, China; ^5^ Organ Transplant Center, Tianjin First Central Hospital, Tianjin, China

**Keywords:** kidney transplant, COVID-19 pneumonia, PAXLOVID, molnupiravir, prognosis

## Abstract

**Objective:**

This retrospective cohort study aimed to assess the clinical features, treatment outcomes, and short-term prognosis in kidney transplant recipients (KTRs) with concurrent coronavirus disease 2019 (COVID-19) pneumonia.

**Methods:**

KTRs with COVID-19 pneumonia who were admitted to our hospital from December 28, 2022, to March 28, 2023 were included in the study. Their clinical symptoms, responses to antiviral medications, and short-term prognosis were analyzed.

**Results:**

A total of 64 KTRs with initial diagnosis of COVID-19 pneumonia were included in this study. The primary symptoms were fever, cough, and myalgia, with an incidence of 79.7%, 89.1%, and 46.9%, respectively. The administration of antiviral drugs (paxlovid or molnupiravir) within 1–5 days and for over 5 days demonstrated a statistically significant reduction in viral shedding time compared to the group without antiviral medication (P=0.002). Both the paxlovid and molnupiravir treatment groups exhibited a significantly shorter duration of viral shedding time in comparison to the group without antiviral drugs (P=0.002). After 6 months of recovery, there was no significantly negative impact on transplant kidney function (P=0.294).

**Conclusion:**

Fever, cough, and myalgia remain common initial symptoms of concurrent COVID-19 pneumonia in KTRs. Early use of antiviral drugs (paxlovid or molnupiravir) is associated with better therapeutic outcomes. Severe acute respiratory syndrome coronavirus 2 (SARS-CoV-2) had a limited impact on the short-term renal function of the KTRs with concurrent moderate or severe COVID-19 pneumonia.

## Introduction

Throughout the duration of the four-year coronavirus disease 2019 (COVID-19) pandemic, the virus has inflicted catastrophic calamities upon individuals worldwide, resulting in the loss of thousands of lives (Karim and Karim, 2021). Through extensive research and the accumulation of clinical cases over this period, efficacious treatment protocols for the general populace have been discerned (Flythe et al., 2021; Massie et al., 2022). Regrettably, in comparison to the general population afflicted by severe acute respiratory syndrome coronavirus 2 (SARS-CoV-2), kidney transplant recipients (KTRs) manifest notably elevated frequencies of grave affliction (44% vs. 6.1%) and mortality (24%–28% vs. 1.4%–4.3%) (Karatas et al., 2021). Unfortunately, our comprehension of the optimal approach to effectively manage COVID-19 infection in KTRs remains limited (Murakami et al., 2023).

As of early 2023, the Omicron variant continues to be the prevailing strain in China. Extensive domestic and international evidence indicates a notable decrease in the pathogenicity of the Omicron variant within the pulmonary system, resulting in a shift in clinical presentations from predominantly pneumonia to upper respiratory tract infections (National Health Commission, 2023). Consequently, our country’s epidemic prevention strategy has been transformed from centralized isolation to a more open approach. However, the specific impact of COVID-19 pneumonia on KTRs, especially in the context of post-treatment monitoring and evaluation, remains understudied.

Given the limited clinical research on this topic, there is an urgent need for new scientific evidence to inform the development of treatment protocols and preventive measures for immunocompromised individuals. This study aims to fill this gap by collecting clinical data from KTRs who contracted COVID-19 pneumonia and were treated at our medical facility between December 2022 and March 2023. The objective is to examine their clinical attributes, diagnostic and therapeutic approaches, and prognosis, thereby furnishing indispensable clinical backing for the formulation of preventive strategies for immunocompromised populations.

## Materials and methods

### Study setting

This retrospective study was conducted at the Second Affiliated Hospital of Hainan Medical University, a large, comprehensive, tertiary Grade A hospital that integrates medical care, teaching, research, rehabilitation, and preventive healthcare. The hospital’s organ transplant center is capable of independently performing transplants of major organs such as the heart, liver, lungs, kidneys, and pancreas, completing approximately 200 organ transplant surgeries each year.

### Study population

Participants were diagnosed with COVID-19 based on a positive reverse transcription-polymerase chain reaction (RT-PCR) test for SARS-CoV-2 from throat swab specimens or next-generation sequencing (NGS) of bronchoalveolar lavage fluid (BALF). Infection severity was categorized as mild, moderate, severe, or critical, with criteria defined as follows (Yang et al., 2020): (1) Mild: no findings of pneumonia on HRCT and presence of mild clinical symptoms; (2) Moderate disease: HRCT manifestations compatible with viral pneumonia and presence of respiratory symptoms and fever; (3) Severe: respiratory rate ≥30 beats per minute or respiratory distress, O_2_ saturation ≤93% in a resting state, or oxygen concentration (FiO_2_) or partial pressure of arterial blood oxygen (PaO_2_) ≤300 mmHg; (4) Critical: respiratory failure requiring mechanical ventilation (MV), shock, or other organ failure requiring ICU admission and monitoring.

### Study design

This retrospective study aimed to statistically analyze demographic data, clinical symptoms, laboratory results, and short-term prognosis of KTRs with COVID-19 pneumonia. The objective was to provide data support for the standardization of treatment for post-transplant COVID-19 pneumonia and to assess the extent of harm to KTRs during the current wave of the pandemic. All KTRs included in this study signed informed consent forms, and the study was approved by the Ethics Committee of the Second Affiliated Hospital of Hainan Medical University (Approval Number: 2024-KCSN-02).

### Treatment plans

The immunosuppression adjustment strategy was based on recommendations from Descartes (Maggiore et al., 2020), while general management strategies were derived from guidelines published by our health ministry (National Health Commission, 2023). Due to limited availability, not all KTRs received antiviral treatment; some were administered paxlovid, and others molnupiravir. The choice of antiviral medication was influenced by availability rather than the severity of infection.

### Observation indicators

Clinical information of KTRs, including the transplantation vintage, gender, age, BMI, number of kidney transplants, vaccine doses, clinical symptoms, SpO2 at admission, antiviral drugs used, pathogen types, underlying diseases, viral shedding time, rejection reactions, and other relevant information were recorded. We also included laboratory indicators at admission, including C-reactive protein (CRP), procalcitonin (PCT), cystatin C, D-dimer (DDP), blood cell counts, and baseline creatinine. RT-PCR assay using the Novel Coronavirus (2019-nCoV) nucleic acid diagnostic kit PCR-Fluorescence Probing (DaAn Gene Co., Ltd, Guangzhou, China) was performed according to the manufacturer’s instructions. Reactions were completed with the Cap Fluorescent Quantitative Polymerase Chain Reaction detection system (FQD-96A). A Ct value less than 35 on either open reading frame (ORF) and/or nucleocapsid protein (N) genes is considered positive.

Stable kidney function was defined as a stable serum creatinine level within the follow-up and no apparent abnormalities in transplant kidney ultrasound. COVID-19 pneumonia was identified by positive pharyngeal swab or BALF NGS tests, combined with typical chest CT imaging changes. Typical chest CT changes for COVID-19 pneumonia included peripheral or peripheral and central ground glass opacities (GGOs) in the early phase. As the disease progressed, GGOs with a crazy-paving appearance and consolidations were evident along with subpleural and parenchymal bands. There was also a predominance of architectural distortion; in the peak stage, these findings had progressed, while the later stage demonstrated their resolution (Brogna et al., 2023).

Follow-up continued until death, graft failure, or the cutoff date (October 31, 2023). This involved monitoring nucleic acid conversion time, serum creatinine, total lymphocyte count, serum albumin, and other factors when the KTRs were admitted to our hospital. Post-discharge, outpatient follow-ups occurred weekly for the first three months, then bi-weekly for the subsequent three months. Comprehensive case data were maintained throughout the follow-up process, with no loss to follow-up.

### Radiographic scores

All patients underwent pulmonary CT examinations when admitted to the hospital (Ai et al., 2020). The pulmonary CT scans were evaluated by two experienced radiologists. Consensus was achieved through discussion in instances of scoring disagreement, and the duration of manual CT scoring completion by the two physicians was documented. In each of the five lung lobes, a CT severity score was assigned based on the degree of involvement: 0 point for no involvement, 1 point for <5% involvement, 2 points for 5–25% involvement, 3 points for 26–50% involvement, 4 points for 50–75% involvement, and 5 points for over 75%. The total CT severity score was the sum of the scores of all five lung lobes, ranging from 0 (no involvement) to 25 (maximum involvement) (Monfared et al., 2021).

### Outcome measures

Allograft dysfunction and death were considered as negative indicators.

### Statistical analysis

Data are presented as mean ± standard deviation (SD) or median ± interquartile range (IQR), according to normality. The continuous variables were compared using t-test or Mann-Whitney U test. Categorical data were compared using the Fisher’s exact test and t -tests, as appropriate. All statistical analyses were conducted using IBM SPSS Version 23. p < 0.05 was considered statistically significant.Significance was adjusted *post hoc*, using Bonferroni’s correction, to reduce the chance of a type I error occurring. The threshold for significance was adjusted from p<0.05 to p<0.0167 as multiple analyses were conducted with effect of antiviral drugs use duration on viral shedding time and efficacy on COVID-19 pneumonia between the different antiviral drugs.

## Results

### Comparison of clinical characteristics between moderate and severe COVID-19 pneumonia patients

This study comprised 64 individuals who had undergone their initial kidney transplant and were diagnosed with concurrent COVID-19 pneumonia. Among these individuals, there were 45 males and 19 females, with ages ranging from 15 to 82 years and an average age of 47.11 ± 13.47 years. The duration between kidney transplantation and onset of COVID-19 pneumonia varied from 1 month to 264 months, with an average of 41.41 ± 48.05 months. It is noteworthy that only 5 cases had received COVID-19 vaccination, while the remaining individuals were unvaccinated. Fever was reported as the first symptom in 51 (79.7%) of the cases. Cough was present in 57 (89.1%) patients, followed by muscle pain in 30 (46.9%) and diarrhea only in 4 patients. Additionally, 30 cases had respiratory distress, accounting for 46.9% of the total. Among the patients, 26 were classified in the severe group, with the remainder classified in the moderate group (n=38). Significant differences were observed in age, CT score, ICU admission or intubation, length of stay, CRP at admission, albumin levels at admission between severe and moderate COVID-19 patients (P<0.05). There were no statistically significant differences in the other indicators. The specific data are indicated in **Table 1**.

**Table 1:** Comparison of clinical features of COVID-19 between moderate disease and severe disease.

	Moderate (n=38)	Severe (n=26)	P value
Gender (Male)	24	21	0.216
Age (years)	43.97±11.92	51.9±14.50	0.023
Interval time between kidney transplantation and onset of COVID-19 pneumonia (months)	39.97±39.49	43.50±59.18	0.776
BMI	21.46±2.67	22.47±3.20	0.179
COVID-19 vaccination	4	1	0.614
Hypertension	21	19	0.237
Diabetes	7	7	0.617
Coronary heart disease	4	6	0.314
CT score	6.84±3.15	15.62±4.10	0.000
WBC on admission (10^9^/L)	5.38±3.22	6.86±3.92	0.104
Lymphocyte counts on admission (10^9^/L)	0.58±0.52	0.61±0.37	0.776
Platelets on admission	186.42±58.54	182.77±72.67	0.825
DDP on admission	0.84±1.55	1. 71±2.07	0.061
CRP on admission (mg/L)	32.60±58.23	71.58±56.49	0.012
PCT on admission (ng/mL)	0.55±1.81	1.89±4.89	0.189
Albumin on admission (g/dL)	39.7±3.7	36.6±6.2	0.014
Baseline creatinine (mg/dL)	179.6±171.1	207.9±151.5	0.498
Viral shedding time (days)	12.9±6.7	17.5±9.9	0.030
Hospital admissions (days)	8.90±3.79	17.54±10.94	0.001
Intubated or admitted to the ICU	0	4	0.024

Data are presented as average ± SD or numbers. WBC, white cell counts; DDP, D-dimer; CRP, C-reaction protein; PCT, procalcitonin.

### Effect of antiviral drugs use duration on viral shedding time

The study evaluated the impact of antiviral drug timing on viral shedding time, corrected by subtracting the duration of drug use from the total shedding time. Out of the 64 participants, 24 did not utilize antiviral drugs, while 10 used them within 1–5 days, and 30 used them for over 5 days. The corrected viral shedding times for these groups were 19.46 (95% CI, 17.19–21.73) days, 12.10 (95% CI, 7.62–16.58) days, and 13.57 (95% CI, 10.82–16.31) days, respectively, indicating a statistically significant difference of three groups (P=0.002). However, there was no statistically significant distinction observed between 1–5 days and over 5 days groups (P=0.539). The three groups demonstrated consistency in various baseline characteristics, including gender, age, transplantation vintage (Interval time between kidney transplantation and onset of COVID-19 pneumonia), hypertension, diabetes, coronary heart disease, BMI, CT score, baseline creatinine, WBC at admission, lymphocyte counts on admission, albumin on admission, CRP, and PCT at admission, with no statistically significant differences observed, as shown in **Table 2**.

**Table 2:** Comparison of clinical features between the patients who did not receive antiviral drugs, used antiviral drugs within 1-5 days and used antiviral drugs over 5 days.

	None (n=24)	1-5 days (n=10)	>5 days (n=30)	P value
Viral shedding time (days)	19.46±5.37	12.10±6.26	13.57±7.34	0.002
Interval time between kidney transplantation and onset of COVID-19 pneumonia (months)	47.54±40.97	25.00±34.62	41.97±56.46	0.465
Gender (Male)	16	7	22	0.932
Hypertension	14	5	21	0.449
Diabetes	6	1	7	0.722
Coronary heart disease	5	3	2	0.139
Age (years)	48.79±14.31	42.20±14.17	47.40±12.59	0.431
BMI	21.73±3.17	21.65±3.37	22.06±2.62	0.889
CT score	9.46±5.60	8.50±4.67	11.80±5.70	0.157
Baseline creatinine (mg/dL)	150.79±102.01	209.47±196.34	217.15±187.63	0.311
WBC on admission (10^9^/L)	5.72±0.36	6.97±5.94	5.86±3.40	0.633
Lymphocyte counts on admission (10^9^/L)	0.50±2.43	0.48±0.23	0.70±0.57	0.239
CRP on admission (mg/L)	49.86±71.14	45.37±44.16	48.67±57.9	0.981
PCT on admission (ng/mL)	1.04±2.49	0.33±0.13	1.39±4.53	0.706
Albumin on admission (g/dL)	38.13±5.16	39.59±4.63	38.25±5.23	0.731

Data are presented as average ± SD or numbers. WBC, white cell counts; CRP, C-reaction protein; PCT, procalcitonin.

### Efficacy on COVID-19 pneumonia between the different antiviral drugs

Among the 64 recipients, 24 individuals did not receive antiviral drugs, 10 received molnupiravir, and 30 received paxlovid. The corrected viral shedding times for these groups were 19.52 (95% CI, 17.15–21.89) days, 14.70 (95% CI, 9.74–19.66) days, and 12.87 (95% CI, 10.28–15.46) days, respectively, displaying a statistically significant difference (P=0.002). The three groups demonstrated consistency in various baseline characteristics, including gender, age, transplantation vintage, hypertension, diabetes, coronary heart disease, BMI, CT score, baseline creatinine, albumin on admission, WBC at admission, CRP, and PCT at admission, with no statistically significant differences observed. The detailed results are shown in **Table 3**.

**Table 3:** Treatment effect of different antiviral drugs on COVID-19.

	None (n=24)	Molnupiravir (n=10)	Paxlovid (n=30)	P value
Viral shedding time (days)	19.52±5.48	14.70±6.93	12.87±7.07	0.002
Interval time between kidney transplantation and onset of COVID-19 pneumonia (months)	47.2±41.9	50.9±88.0	101.8±206.9	0.490
Gender (Male)	16	6	23	0.500
Hypertension	14	8	18	0.539
Diabetes	6	1	7	0.722
Coronary heart disease	5	0	5	0.386
Age (years)	48.35±14.47	45.60±16.07	46.68±12.16	0.488
BMI	21.54±3.10	21.86±3.66	22.12±2.57	0.764
CT score	9.52±3.10	13.00±5.56	10.23±5.45	0.307
Baseline creatinine (mg/dL)	153.74±103.25	223.40±190.32	208.33±187.78	0.765
WBC on admission (10^9^/L)	5.79±2.45	6.80±3.91	5.6±4.17	0.793
CRP on admission (mg/L)	51.84±72.16	74.18±77.23	37.27±39.70	0.401
PCT on admission (ng/mL)	1.08±2.54	1.24±2.78	1.05±4.23	0.995
Albumin on admission (g/dL)	38.04±5.26	38.30±4.84	38.73±5.13	0.400

Data are presented as average ± SD or numbers. WBC, white cell counts; CRP, C-reaction protein; PCT, procalcitonin; Cr, creatinine.

### Pathogen spectrum of KTRs with COVID-19 pneumonia

Upon admission, a total of 31 recipients underwent NGS testing. Among these recipients, 30 specimens were obtained from BALF, one from blood, and another from sputum. Bacterial infections were identified in 20 specimens, including *Staphylococcus argenteus*, *Acinetobacter baumannii*, *Pseudomonas aeruginosa*, *Tropheryma whipplei*, *Klebsiella pneumoniae*, *Streptococcus pneumoniae*, *Enterococcus faecalis*, *Escherichia coli*, *Hemophilus influenzae*, *Pseudomonas maltophilia*, and *Legionella pneumophila*. Additionally, fungal infections, including *Aspergillus flavus*, *Candida albicans*, *Pneumocystis jirovecii*, *Aspergillus fumigatus*, *Candida parapsilosis*, and *Candida tropicalis*, were detected in 14 specimens. Furthermore, 11 specimens exhibited co-infection with Human betaherpesvirus 5. Notably, a significant proportion of recipients, accounting for 77.4% (24 out of 31), presented with mixed infections, involving bacteria or fungi in conjunction with COVID-19 pneumonia.

### Short-term prognosis of KTRs with COVID-19 pneumonia

Four patients died within 6 months of follow-up, and 1 had kidney allograft dysfunction. A comparison of baseline creatinine (176.63 ± 149.62 μmol/L) and creatinine at 6 months (153.46 ± 76.25 μmol/L) in the remaining 59 recipients showed no statistically significant difference (P=0.173).

## Discussion

The COVID-19 pandemic, now spanning over four years, has seen a reduction in virulence due to the emergence of less pathogenic variants like Omicron. Despite the World Health Organization’s (WHO) announcement on May 5, 2023, that the pandemic no longer qualifies as a global public health emergency, the virus is expected to persist and continue affecting the human population. The prognosis of KTRs in relation to SARS-CoV-2 remains a relatively unexplored area, and our study aims to contribute to this knowledge gap.

### Clinical data and symptoms

Our study reveals that fever (79.7%) and cough (89.1%) persist as the most prevalent clinical symptoms of post-transplant COVID-19 pneumonia, with an elevated occurrence compared with general population and exclusively COVID-19 infected KTRs (Belsky et al., 2021; Monfared et al., 2021). Furthermore, this study demonstrates higher incidence rates of muscle pain and shortness of breath compared to previous literature reports (Akalin et al., 2020). The different results may be attributed to the differences in patients enrollment and population selection. In our study, the incidence of diarrhea was 6.25%, which aligns with the findings of *Monfared* et al (Monfared et al., 2021). All participants in our study discontinued Mycophenolate Mofetil (MMF) and either reduced or stopped Calcineurin Inhibitors (CNI) based on the severity of infection. The duration of immunosuppressive drug discontinuation ranged from 6 to 33 days, with a median of 6 days (refer to **Supplementary Materials** for further details). However, no instances of acute rejection were observed in KTRs, potentially attributed to the increased utilization of corticosteroids during COVID-19 pneumonia treatment and the compromised immune systems of the patients (Maggiore et al., 2020; Monfared et al., 2021).

In our short-term follow-up of 64 KTRs with COVID-19 pneumonia, we observed no adverse events such as pulmonary embolism, possibly due to adequate administration of anticoagulant therapy and outpatient health education (Basic-Jukic et al., 2021). There were 1 patient had kidney allograft dysfunction, three died of COVID-19 pneumonia, and 1 dead of aspergillosis, resulting in an overall mortality and allograft dysfunction rate of 7.81% (5/64). Our findings suggest that personalized discontinuation of immunosuppressive drugs can be considered a safe and dependable approach for KTRs. In the present study, a significant statistical disparity was observed in the utilization of lung CT scans for the evaluation of moderate and severe COVID-19 pneumonia (P<0.05), thereby reinforcing the favorable contribution of lung CT scans in the assessment of this condition (Fields et al., 2020). Our data indicate no statistically significant difference (P=0.148) between the baseline mean serum creatinine levels (174.8 ± 150.3 μmol/L) and those at six months after discharge (149.9 ± 71.8 μmol/L), suggesting no detrimental impact of SARS-CoV-2 on transplant kidney function over six months. However, a marginal decline in mean serum creatinine levels at the six-month after discharge was observed, potentially attributable to compromised immunity in individuals previously afflicted with COVID-19 pneumonia (Zhu et al., 2020a; Cristelli et al., 2021), as well as reduced doses of CNI medications (Zhu et al., 2020b).

### Association of severe pneumonia with worsened transplant kidney function

Our investigation revealed no noteworthy disparity in baseline creatinine levels between the moderate and severe COVID-19 pneumonia cohorts (P>0.05), aligning with the findings of Malinowska’s study (Malinowska et al., 2022). This suggests that the severity of SARS-CoV-2 does not exert an immediate influence on transplant kidney function, but rather manifests long-term consequences.

### Impact of antiviral drug duration and efficacy

Our study revealed a significant correlation between the timing of antiviral drug administration and the effectiveness of treatment in KTRs with COVID-19 pneumonia (Hung et al., 2022; Sun et al., 2022). Individuals who received antiviral drugs within 1–5 days exhibited a shorter duration of viral shedding (12.10 days) compared to those who received antiviral drugs for over 5 days (13.57 days) or did not receive them at all (19.46 days). Both Paxlovid and Molnupiravir demonstrated similar efficacy in reducing viral shedding (12.87 ± 7.07 days vs. 14.70 ± 6.93 days), with Paxlovid exhibiting superiority over non-users (P<0.0167) (Park et al., 2023). However, the group receiving molnupiravir exhibited a shorter adjusted duration of viral shedding in comparison to the non-user group (14.70 ± 6.93 days vs. 19.52 ± 5.48 days), though this disparity did not reach statistical significance (P=0.055), likely due to the small sample size. During our short-term follow-up, we did not observe any immediate impact of antiviral medications on the functioning of the transplanted kidney, which aligns with the findings of Wen et al.’s investigation (Wen et al., 2022). Our data indicate that the early administration of antiviral drugs produces more favorable outcomes (Cegolon et al., 2023; Saravolatz et al., 2023).

### Antibiotic use in KTRs with COVID-19 pneumonia

The utilization of antibiotics in KTRs with COVID-19 pneumonia has been a subject of debate (Maggiore et al., 2020). In this retrospective investigation, we employed NGS technology to analyze a total of 31 specimens, revealing a notable prevalence of mixed infections (77.4%, 24/31), significantly higher than the general population rate of 26% (Pegoraro et al., 2023). The predominant bacteria were *Escherichia coli*, *Staphylococcus aureus*, *Klebsiella pneumoniae*, *Pseudomonas aeruginosa*, *Acinetobacter baumannii*, and *Streptococcus pneumoniae* (Antimicrobial Resistance C, 2022) Invasive fungal infections were also increasingly reported (Kanj et al., 2023). Mixed infections appear more common, emphasizing the necessity of judicious antibiotic prophylaxis. In comparison to conventional lung CT scans, the utilization of NGS detection enables the early detection of co-infections (Xu et al., 2023), thereby facilitating accurate clinical intervention and mitigating the inappropriate use of antibiotics. Regrettably, NGS does not encompass susceptibility testing, a feature commonly found in traditional pathogen detection methods. Nonetheless, the rapid and cost-effective generation of genome-scale sequence data associated with NGS presents a more comprehensive pathogen profile, thereby advancing our comprehension of real-world scenarios (Xuan et al., 2013).

Limitations exist with our study. First, this is a single-center, retrospective cohort study with a small sample size. Larger scale prospective studies are required to verify our findings. Second, the follow-up period was only six months, longer follow-ups and observations should be conducted in the future. Finally, the retrospective nature of the study introduced certain biases in the patient cohort structure.

Notwithstanding these constraints, our study demonstrates valuable insights into the management of KTRs with COVID-19 pneumonia. We observed that antiviral medications exhibit efficacy regardless of the disease stage, the early administration of prophylactic antibiotics is of utmost importance, and no infections or detrimental effects on graft function resulting from antiviral drugs were detected six months after recovery.

## Data Availability

The datasets presented in this study can be found in online repositories. The names of the repository/repositories and accession number(s) can be found in the article/**Supplementary Material**.
