# Supplementary material for: Clinical features and outcomes in kidney transplant recipients with COVID-19 pneumonia: a single center retrospective cohort study
Source: Front Cell Infect Microbiol. 2024 Aug 15;14:1392491. doi: 10.3389/fcimb.2024.1392491 (PMC11358093; doi:10.3389/fcimb.2024.1392491)
Supplement: Supplementary file 1 [file Table1.docx]

**COVID-19 PCR Testing**

Clinical specimens for COVID-19 diagnostic testing were obtained following the Centers for Disease Control and Prevention (CDC) guidelines. Nasopharyngeal swabs were collected and placed into 3 mL viral transport media. RNA extraction was then conducted, followed by real-time RT PCR to amplify two distinct regions within SARS-COV-2 (Luminex targets ORF1a/N, Abbott targets RdRp/N, and Hologic focuses on separate ORF1a regions) using one of three commercial methods: Luminex ARIES, Abbott m2000, or Hologic Panther Fusion SARS-COV-2 assays. All testings were performed in adherence to the manufacturer’s instructions.

**Treatment strategies for COVID-19 kidney transplant patients**

***Adjustment of immunosuppressant strategy***

If the lung CT of patients shows positive or highly suspicious manifestations, but the extent of the lesions is mild and the lung involvement is less than 10%, the dosage of anti-metabolite drugs can be reduced or discontinued, and the dose of CNI drugs can be appropriately reduced or maintained at a normal level. While if the lung CT shows typical positive manifestations, but the lung involvement is less than 5 points and the patient’s condition is stable, the anti-metabolite drugs can be discontinued, and the dose of CNI drugs can be appropriately reduced. For patients with severe conditions and the lung CT shows typical and extensive positive manifestations, with lung involvement greater than 30%, all immunosuppressants should be discontinued if necessary. Due to the significant increase in the serum drug concentration of tacrolimus by the use of nivolumab, all immunosuppressants, except steroids, should be temporarily suspended regardless of the severity lung infection. The dosage reduction, discontinuation, and recovery of immunosuppressants should be determined based on the progression of lung CT positive manifestations, improvement of clinical symptoms, and use of methylprednisolone.

***Oxygen therapy***

The goal of oxygen therapy is to achieve a blood oxygen saturation level of more than 95%. Oxygen therapy is needed for patients with hypoxemia. It can be intensified to dual-channel (mask+nasal cannula) oxygen therapy if necessary. When patients develop respiratory distress and standard oxygen therapy is ineffective, high-flow nasal cannula oxygen therapy (HFNO) or non-invasive ventilation (NIV) can be chosen. Patients receiving HFNO and NIV therapy need to be closely monitored, and if the patient deteriorates severely or fails to improve within a short period of time, tracheal intubation should be considered.

***Glucocorticoid therapy***

The dosage of methylprednisolone or dexamethasone sodium phosphate injection should be determined based on the patient’s temperature control, with the principle of using the minimum dosage to keep the patient’s temperature below 37.5°C. For patients with early lung CT showing typical ground-glass opacities with or without fever, the initial dose of methylprednisolone can be 20mg qd, gradually increasing to 20mg q12h according to changes in the condition. For patients with high fever, multifocal or large patchy ground-glass opacities in both lungs, the initial dose of methylprednisolone can be increased to 40mg q12h. If the temperature cannot be controlled, a small amount of dexamethasone can be given intermittently to assist with fever reduction. The treatment course with methylprednisolone generally does not exceed 14 days.

***Antimicrobial therapy***

For patients with an early course of the disease and no clear evidence of bacterial infection, prophylactic use of antibiotics can be considered. For patients hospitalized for more than 1 week, it is recommended to use antibiotics based on microbiological results and specific circumstances, and consider prophylactic use of antifungal drugs.

***Supportive therapy***

Supportive therapy is particularly important in the treatment of post-transplant patients. In addition to ensuring adequate energy intake and maintaining internal stability such as water, electrolytes, and acid-base balance, timely administration of appropriate doses of immunoglobulin (5-10g qd) and albumin is necessary. During the entire course of the disease, there is a significant metabolic consumption, and most patients experience hypoalbuminemia. Timely supplementation of albumin and the use of appropriate diuretics can help reduce pulmonary interstitial exudation. Therefore, based on changes in the patient’s condition and laboratory indicators during the course of the disease, sufficient albumin (20g qd/bid) should be given as much as possible.

***Anticoagulant therapy***

Prophylactic anticoagulant therapy should be administered to hospitalized patients without contraindications. Low molecular weight heparin is the first choice, followed by aspirin, clopidogrel, *etc*.

***Pulmonary rehabilitation therapy***

Pulmonary rehabilitation therapy, including prone positioning ventilation therapy, should be performed for all COVID-19 patients.

***Antiviral therapy***

Nirmatrelvir/Ritonavir (Paxlovid) is currently recommended for use within 5 days of symptom onset, and its use beyond 5 days is beyond the indications. Paxlovid significantly increases the blood drug concentration of immunosuppressants. In the early course of post-kidney transplant patients, when lung symptoms are not severe and immunosuppressants have not been discontinued, the use of Paxlovid is generally not prioritized. However, when the patient’s condition progresses and severe lung symptoms appear, requiring complete discontinuation of immunosuppressants, the onset of the disease has often exceeded 5 days. Nevertheless, we still recommend that eligible post-kidney transplant patients, under the monitoring of transplant physicians, use Paxlovid as early as possible, even if the optimal treatment window of 5 days has passed, under the condition of complete discontinuation of immunosuppressants or close monitoring of immunosuppressant blood concentrations. If the baseline creatinine is less than 200 μmol/L, the dosage is as follows: Nirmatrelvir 300 mg and Ritonavir 100 mg (Paxlovid) are taken simultaneously, once every 12 hours for 5 consecutive days. If the baseline creatinine is greater than 200 μmol/L, the dosage is as follows: Nirmatrelvir 150 mg and Ritonavir 50 mg (Paxlovid) are taken simultaneously, once every 12 hours for 5 consecutive days. The dosage of Molnupiravir is 0.8 g every 12 hours for 5 consecutive days.

**Data Collection**

For each confirmed COVID-19 kidney transplant patient, the number of COVID-19 vaccine doses administered was obtained from the patient or their family. The other clinical data was collected from electronic medical records. The information including age, gender, body mass index, number of vaccine doses received, hypertension, diabetes, cardiovascular history, and kidney transplant-related information were collected to describe the characteristics of the patients.

For each patient, we collected data on the time of kidney transplantation, clinical symptoms and signs, spO2 within 24 hours of admission, white blood cell count, platelet count, lymphocyte count, CD3, CD4, CD8 cell counts, ferritin, C-reactive protein, procalcitonin, D-dimer, blood creatinine, cystatin C, troponin, and creatine kinase. We also collected data on baseline vital signs within 24 hours of admission, peak creatinine level, treatment and prevention measures, chest X-ray and chest CT scan results, microbiological examinations, pathogen identification, antibiotic treatment, pneumonia severity index (if respiratory rate data were missing, we assumed it was normal), and cardiovascular history corresponded to a history of coronary artery disease or documented heart failure.

Creatinine baseline was defined as the rounded average of COVID-prior-year results. Proteinuria (>1g/24h) was determined based on at least 2 samples. Bronchoscopic sampling included bronchoalveolar lavage (BAL) and bronchial washing. Acute kidney injury (AKI) was defined based on the highest creatinine value during the stay according to the KDIGO definition. Immunosuppressive reduction referred to the discontinuation of at least one immunosuppressive treatment.

Multidrug-resistant (MDR) bacteria were defined according to the Centers for Disease Control and Prevention (CDC) definition as follows: *Enterobacteriaceae* non-susceptible to at least 1 agent in more than 3 antimicrobial categories (Penicillins, Cephalosporins, Aminoglycosides, Fluoroquinolones, Carbapenems), Methicillin-resistant *Staphylococcus aureus* (MRSA), and *Pseudomonas aeruginosa* resistant to at least one agent in 3 or more antibiotic classes (antipseudomonal Cephalosporins and Fluoroquinolones, Aminoglycosides).

**Pathogen Identification**

Positive etiological diagnosis was confirmed under the following conditions: the presence of recognized Gram-positive or Gram-negative bacteria by culture using blood, sputum, bronchial washings, or bronchoalveolar lavage. The identification of the COVID-19 virus was based on a positive PCR test of oral and pharyngeal swabs. RNA and DNA pathogens were also detected by Next-generation sequencing (NGS) of bronchoalveolar lavage and blood specimens.
